# Anticancer Effect of Rh2, a Histone Deacetylase Inhibitor, in HepG2 Cells and HepG2 Cell-Derived Xenograft Tumors Occurs via the Inhibition of HDACs and Activation of the MAPK Signaling Pathway

**DOI:** 10.31557/APJCP.2021.22.8.2529

**Published:** 2021-08

**Authors:** Shi Qing Qiang, Gao Chao Qin, Li Jing, Feng Zi Qiang, Qin Hong Mei, Chen Di Long

**Affiliations:** 1 *The Second Affiliated Hospital of Chongqing Medical University, Chongqing, P.R. China. *; 2 *Laboratory of Stem Cell and Tissue Engineering, Department of Histology and Embryology, Chongqing Medical University, Chongqing, China. *; 3 *Department of Pharmacy, Renhe Community Health Service Center of Chongqing Liangjiang New Area, Chongqing, China. *; 4 *The First People’s Hospital of Chongqing Liangjiang New Area, Chongqing, China. *; 5 *Chongqing Three Gorges Medical College, Chongqing, P.R. China. *

**Keywords:** 20(S)-ginsenoside Rh2, HepG2 cells, histone deacetylase, MAPK, hypoxia-inducible factor

## Abstract

**Purpose:**

To investigate the effect of 20(S)-ginsenoside Rh2 (Rh2) on anti HepG2 liver cancer cells and HepG2 cell-derived xenograft tumors, and explore the underlying mechanisms.

**Materials and Methods:**

The activity of total HDACs and HAT were assessed with a HDACs colorimetric kit. Expression of HDAC1, HDAC2, HDAC6, p-ERK, ERK, p-P38, P38, p-JNK and JNK proteins was tested by Western blotting.H3K9 and H3K14 proteins were also checked by immunofluorescence, changes in cell cycle distribution with flow cytometry, cell apoptosis with annexin V-FTIC/PI double staining. Activity of Renilla luciferase (HIF) was detected using the Luciferase Reporter Assay system reagent. Gene expression for CyclinD1, Bcl-2, Bax, HIF, IL-1, IL-6, IL-10 and TNF-α was tested by q-PCR. Expression levels of CD31 and Ki-67 was tested by immunohistochemical staining.

**Results:**

Total HDAC activity was decreased and total histone acetyltransferase (HAT)activity was increased in a time-dependent manner. Expression of HDAC1 and p-JNK proteins was significantly increased, expression levels of p-ERK was decreased. H3K9 and H3K14 fluorescence protein were increased. Flow cytometric analysis of the cell cycle revealed that the percentage of cells in the G0/G1 phase in the treatment group(64.35±1.36%) was significantly increased compared with the untreated group(61.61±1.23%).The apoptotic rate of the HepG2 group was 10.03±1.92%, which increased to 17.87±1.67% in the treatment group. Expression levels of the transcription factor HIF were also increased in HepG2 cells following induction by Rh2. Expression of CyclinD1 and Bcl-2 at the genetic level was significantly decreased, while expression levels of Bax, HIF, IL-1, IL-6, IL-10 and TNF-α was increased. In vivo, the expression levels of both CD31 and Ki-67 proteins were significantly down-regulated in the treatment group compared with the control group.

**Conclusions:**

The effects of Rh2 were suggested to occur through the inhibition of total HDAC activity, which subsequently induced MAPK signaling and down-regulated the expression of HIF.

## Introduction

Liver cancer is a common malignant tumor in developing countries (Fujiwara et al., 2018; Kulik and El-Serag, 2019). Surgical resection is currently the most effective treatment option for liver cancer; however, only a small number of patients are eligible for surgery and the rate of recurrence remains high (Tsai et al., 2014). For the majority of cases, liver cancer is only diagnosed at the advanced stages. To date, the understanding of the underlying mechanisms of liver cancer occurrence and development is limited and therefore, current therapeutic regimens are unsatisfactory (Lu et al., 2018a). Palliative therapy for liver cancer is limited because liver cancer is often resistant to conventional chemotherapeutics (Pinter and Peck-Radosavljevic, 2018). Thus, discovering effective therapies for liver cancer remains a priority. Current research on this disease is focusing on identifying novel drugs that will enhance the effects of chemotherapy (Pinter and Peck-Radosavljevic, 2018). 

Previous studies have reported that the over-expression of histone deacetylases (HDACs) affected the progression of liver cancer (Zhang et al., 2019b). HDACs comprise four highly conserved families of enzymes, which are recruited to multi-protein transcription complexes on the chromosome and act as epigenetic transcriptional co-inhibitors to suppress target gene transcription(Lu et al., 2018a). An increasing number of studies have demonstrated that the occurrence and development of cancer was significantly affected by epigenetic mechanisms (Vialou et al., 2013; Adams and Eischen, 2016; Gao et al., 2018). For example, a previous study found that HDACs played a number of important roles in cancer, including decreasing chemotherapy resistance and increasing the levels of apoptosis (Gao et al., 2018). HDACs were suggested to represent a potential target for pharmacological inhibition, as abnormal epigenetic changes have been established to be characteristic of cancers (Mrakovcic et al., 2017). Therefore, the use of HDAC inhibitors is becoming increasingly popular for cancer treatment and has been predicted to be successful in preclinical settings.

20(S)-ginsenoside Rh2 (Rh2) is a Traditional Chinese medicine that has demonstrated antitumor properties in malignant liver cancer through its ability to repair DNA damage, regulate cell apoptosis, and inhibit proliferation and immune regulation, amongst other mechanisms(Lu et al., 2018b; Lee et al., 2019; Xiao et al., 2019). Previous studies have also reported that Rh2 can regulate antitumor mechanisms by targeting the MAPK signaling pathways (Yoon et al., 2012; Tang et al., 2013). Recent research found that Rh2 activated NF-κB by inhibiting mitogen-activated protein kinase kinasekinas 7 phosphorylation and lipopolysaccharide-induced accumulation of hypoxia-inducible factor(HIF), providing a mechanistic insight into the potential therapeutic role of Rh2 for HIF-dependent tumors (Guan et al., 2018; Xiao et al., 2019). A previous study demonstrated that HIF up-regulated the expression levels of VEGF in the breast tumor to affect angiogenesis (Su et al., 2012; Gao et al., 2017).To effectively treat the tumor, it is necessary to understand the molecular mechanisms underlying the formation of new blood vessels (Mendes et al., 2014). However, whether Rh2 can exerts inhibitory effects over HDACs in HepG2 liver cancer cells and HepG2 cell-derived xenograft tumors and subsequently regulate the expression of downstream MAPK signal pathway and tumor angiogenesis remains to be determined. 

## Materials and Methods

Cell lines and culture. Frozen stocks of HepG2 cells (Gaining Biological Technology Co., Ltd; Shanghai) were obtained from the Laboratory of Histology and Embryology, Cells were cultured with 10% standard FBS (HyClone; Cytiva), and maintained at 37˚C with constant humidity.

Nude mouse xenograft model. All experiment animals in this study were housed in an Association of Assessment and Accreditation of Laboratory Animal Care Internation(AAALAC) certified animal facility in our university with temperature and light cycles(24˚C and 12/12 light),with sterile mouse chow and water ad libitum. Experiment procedure were performed in pathogen-free. Nude BAIL/c mice, 6-to 8-weeks-old and 20-to 25-g-weight, were obtained from The Laboratory Animal Center of Chongqing Medical University (Chongqing, China). 15 of nude mice were divided into three groups according to completely randomized design: Untreated, HepG2 and HepG2 + Rh2. A total of 5x10^7^ HepG2 cells + normal saline were subcutaneously injected into each mouse in the HepG2 and HepG2 + Rh2 group, while 0.1 ml normal saline was injected into mice in the untreated group. Upon the tumors reaching 0.5cm, the HepG2 + Rh2 group mice were fed with 20 mg/kg Rh2 for 20 consecutive days by oral gavage. While untreated and HepG2 group were fed with normal saline for 20 consecutive days by oral gavage. The diameter of the tumor and the weight of each mouse were recorded every day. At the end of the experiment, the mice were anesthetized with60 mg/kg sodium pentobarbital and euthanized with 100 mg/kg intravenous potassium chloride (Hawkins et al., 2016). All animal experimental protocols were approved by the Animal Ethics Committee of Chongqing Medical University (approval no.JN.No20200115S0640120). 

Antibodies and chemicals. Rh2 was provided by the National Standard Network.DMSO and Cell Counting Kit-8 (CCK-8) reagent were purchased from TakaraBio, Inc.The Annexin V-FITC Apoptosis Detection kit was obtained from Nanjing KeyGen Biotech Co., Ltd. Anti-HDAC1 (1:1,000), anti-HDAC2 (1:1,000) and anti-HDAC6 (1:100) primary antibodies were purchased from AB Antibody Technology. Anti-ERK(1:1,000), phosphorylated (p)-ERK(1:1,000), anti-P38(1:1,000), anti-p-P38 (1:1,000), anti-JNK(1:1,000) and anti-p-JNK(1:1,000) primary antibodies were purchased from Sigma-Aldrich; Merck KGaA. HDAC and histone acetyltransferases (HAT) ELISA kits were obtained from Cell Signaling Technology, Inc. HRP-conjugated goat anti-rabbitIgG and anti-mouse IgG secondary antibodies were purchased from Beyotime Institute of Biotechnology. 

Cell cycle analysis.Flow cytometrywas used to analyze the distribution of the cell cycle according to the method described in our previous study (Shi et al., 2016).

Flow cytometric analysis of apoptosis.Cell apoptosis was analyzed using a BD FACScanflow cytometer (BD Biosciences) accordingto the method described in our previous study (Shi et al., 2016).

Transient transfection and relative luciferase activity determination. To determine the Renilla luciferase activity in the cell supernatant, cells were divided into four groups: i) Positive control group, in which cells were transfected with a plasmid(pad-track-tox); ii) false positive group, in which cells were transfected with liposomes; iii) HIF group, in which cells were transfected with a plasmid encoding Renilla luciferase (HIF); and iv) HIF+Rh2 group, in which cells were transfected with HIF and treated with Rh2 for 6, 12, 24 or 48h.Renillaluciferase activity was measured in the supernatant according to the manufacturer’s protocol (Takara Bio, Inc.) (Shi et al., 2014).

Immunofluorescence assay. Briefly, 1x10^7^cells were plated onto a sterile glass slide in a six-well plate. Following 24h of incubation, cells were either treated with 80 µmol/l Rh2 (treatment group) or DMSO (untreated group) for a further 24h. After treatment, cells were fixed with 4% paraformaldehyde and permeabilized with0.3% Triton X-100. Then, cells were blocked with goat serum (HyClone; Cytiva) prior to incubation with an anti-histone 3 lysine 14 (H3K14; 1:1000) or anti-histone 3 lysine 9 (H3K9; 1:1000) primary antibody. Following an overnight incubation, cells were incubated with an anti-rabbit fluorescent secondary antibody or 1h, stained with propidium iodide (PI; Beyotime Institute of Biotechnology), mounted with 50% glycerol andvisualized using afluorescent microscope(Shi et al., 2016).

ELISA.ELISAkits were used to determine the activities of HDACs and HATs, according to the manufacturers’protocols (Shi et al., 2016).

Immunohistochemistry.Immunocytochemistry was performed to determine the subcellular localization of CD31 and Ki-67 using an immunocytochemical kit (Cell Signaling Technology, Inc.;), according to the manufacturer’s protocol (Shi et al., 2016).

Western blotting. Total protein was extracted and protein concentration was determined using a Bradford assay(Bio-Rad Laboratories, Inc.). Then, 20-30µg protein/lane was separated via 10-15% SDS-PAGE. The separated proteins were subsequently transferred on to PDVF membranes and blocked. The membranes were then incubated with primary antibodies at the suitable aforementioned dilutions. Following the primary antibody incubation, the membranes were incubated withHRP-conjugated goat anti-rabbit IgG or goat anti-mouse IgG (1:2,000;Bio-Rad Laboratories, Inc.) secondary antibodies.Protein bands were visualized using an Immun-Star™ HRP Chemiluminescent kit on aVersaDoc™ imaging system (Bio-Rad Laboratories, Inc.),which also performed the densitometric analysis. 

Reverse transcription-quantitative PCR (RT-qPCR).Total RNA was extracted from cells using TRIzol®reagent (Invitrogen; Thermo Fisher Scientific, Inc.). Total RNA was reverse transcribed into cDNA usingMMLV transcriptaseand Oligod(T) primers. qPCR was subsequently performed using a25µl reaction system on a Real-Time PCR system (Applied Biosystems; Thermo Fisher Scientific, Inc.). The following primer sequences were used for the qPCR: IL-1forward, TGAAAGCTCTCCACCTCCAGGGACA.andreverse,GAGGCCCAAGGCCACAGGTATTTTG;IL-6forward,CCTCACCCTCCAACAAAGATandreverse,GCCTCAGACATCTCCAGTCC; IL-10forward, TGCCTTCAGCAGAGTGAAGA and reverse, GTCTTGGTTCTCAGCTTGGG; HIF forward,AGCATGTAGACTGCTGGGGCAA and reverse, CCTGCAGTAGGTTTCTGCTGCCTTG; and TNF-α forward, CCTGTAGCCCATGTTGTAGCA and reverse,TTGAAGAGGACCTGGGAGTAG. The thermocycling conditions for the qPCR were set according to the manufacturer’s protocol. β-actin was used as the reference gene and the fold change in mRNA expression levels was quantified using the 2^-ΔΔCq^ method.

Statistical analysis. Statistical analysis was performed using SPSS 21.0 software (IBM Corp). Statistical differences between groups were determined using a Student’s t-test. All experiments were repeated at least in triplicate, with three independent repeats per experimental condition. P<0.05 was considered to indicate a statistically significant difference. 

## Results

*Rh2 inhibits the activity of HDACs and increases histone acetylation in HepG2 cells*

**Figure 1:**
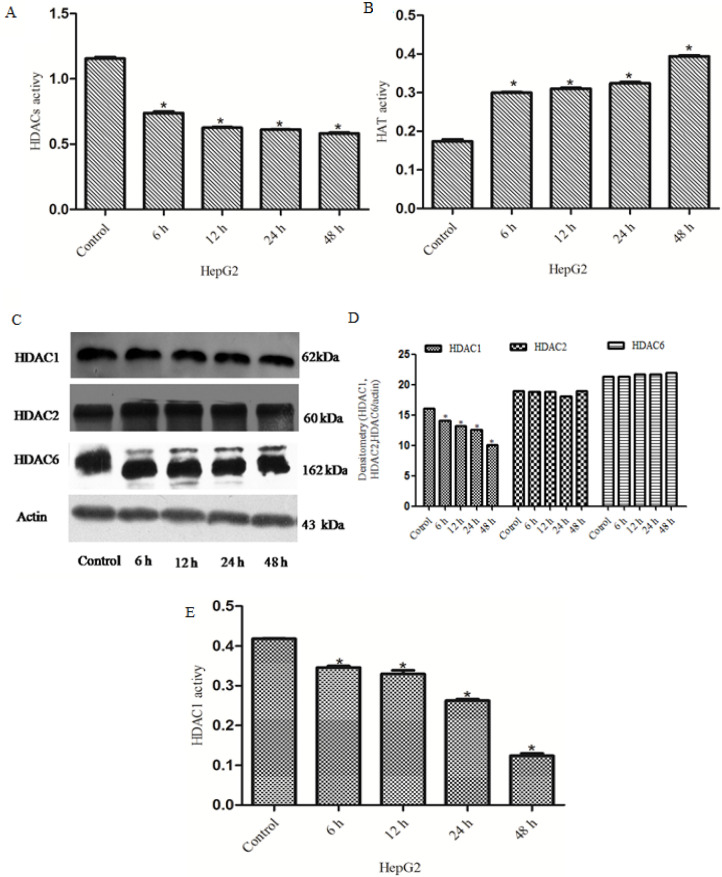
Rh2 Inhibits the Activity of HDACs and Increases the Activity of HATs. ELISAs were Used to DetermineHDAC and HAT Activity. (A) HDAC activity decreasedin a time-dependent manner in the treatment group. (B) HAT activity was increased in the treatment group. (C) Expression levels of HDAC1, HDAC2 andHDAC6 were analyzed using western blotting. β-actin was used asthe referenceprotein.(D) HDAC1 activity was analyzed using an ELISA. All experiments were repeated at least three times.Rh2, 20(S)-ginsenoside Rh2; HDAC, histone deacetylase; HAT, histone acetyltransferase

**Figure 2:**
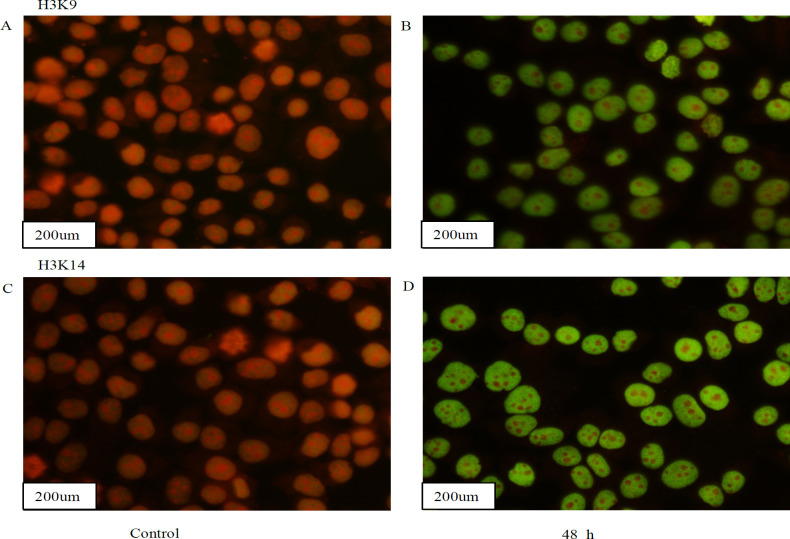
Rh2 Increases Histone Acetylation. (A-D)Cells were incubated with Rh2 for 48h,and ﬂuorescence microscopy was used to analyze the localization of H3K9 andH3K14 in HepG2 cells (magnification, x400;scale bar, 200-μm). All experiments were repeated at least three times. Rh2, 20(S)-ginsenoside Rh2; H3K9, histone 3 lysine 9; H3K14, histone 3 lysine 14

HDACs have been associated with and found to affect the development of numerous types of cancer. HDACs exert the opposite effects to HATs by removing acetyl groups from histone lysine tails, which can stimulate biological processes such as gene transcription (Lernoux et al., 2018; Eto et al., 2019). Previous clinical studies have demonstrated that the expression levels of HDACs were up-regulated in liver cancer tissues (Venturelli et al., 2013; Venturelli et al., 2019). The present study aimed to investigate whether Rh2exerted pharmacological effects against HDACs in liver cancer by acting as a HDAC inhibitor. Total HDAC activity was decreased and total HAT activity was increased in a time-dependent manner in the treatment group (Figure 1A and B). The protein expression levels of HDAC1, HDAC2 and HDAC6 were also analyzed; Compared with the control group, the protein expression levels of HDAC1were down-regulated, while no significant differences were observed in the protein expression levels of HDAC2 and HDAC6 (Figure 1C and D). ELISAs were performed to determine the activity of HDAC1; the results revealed that the activity of total HDAC1 was decreased in a time-dependent manner in the treatment groups (Figure 1E). Immunoﬂuorescence analysis was used to determine the localization of H3K9 and H3K14. As shown in Figure 2, a bright green ﬂuorescent signal was observed in the nuclei of HepG2 cells in the treatment group, while little to no green ﬂuorescent signal was observed in the nuclei ofHepG2 cells in the untreated group. These results suggested that Rh2 may inhibit the activity of HDACs, as a HDAC inhibitor, to alter histone acetylation.

*Rh2 activates the MAPK signaling pathway and down-regulates the expression levels of HIF*

**Figure 3:**
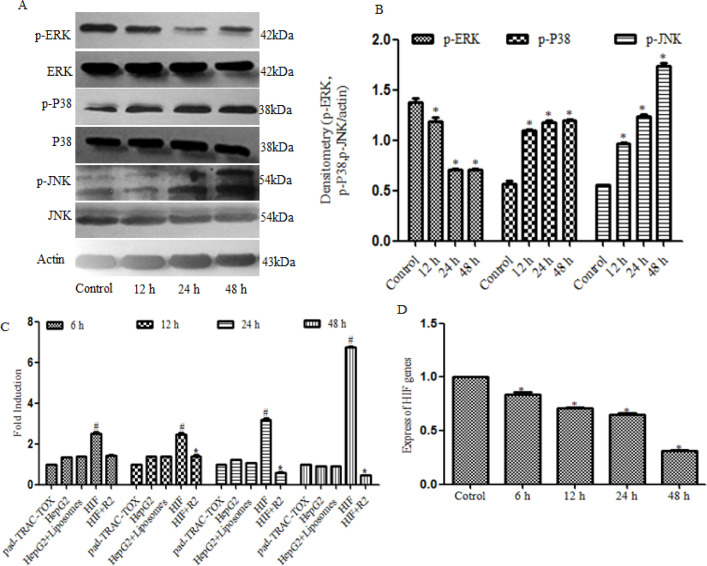
R2 Up-Regulates the Expression Levels of MAPK Signaling Pathway Proteins and Down-Regulated the Expression Levels of HIF. (A and B) HepG2cells were treated with Rh2 for 12,24 or 48h, and western blotting was used to analyze the expression levels of p-ERK,ERK,p-P38,P38,p-JNK and JNK.β-actin was used as the referenceprotein. (C) Activity of Renilla luciferase (HIF) was detected using the Luciferase Reporter Assay system reagent. (D) Reverse transcription-quantitative PCR was used to determine the mRNA expression levels of HIF. All experiments were repeated at least three times.*P<0.05 vs. control. Rh2, 20(S)-ginsenoside Rh2; p-, phosphorylated; HIF, hypoxia-inducible factor

Whether Rh2 could activate the MAPK signaling pathway and regulate the expression of downstream transcription factors was further investigated through analyzing the expression levels of p-ERK, ERK, p-P38, P38, p-JNK and JNK. The results revealed that the protein expression levels of p-P38 and p-JNK were significantly up-regulated in the treatment group compared with the untreated group, while the protein expression levels of p-ERK were down-regulated in the treatment group compared with the untreated group(Figure 3A and B). Furthermore, to determine the activity of downstream transcription factors, Renilla luciferase activity was measured in the cells upernatant. Rh2 was discovered to down-regulate the expression levels of the HIF transcription factor (Figure 3C). RT-qPCR analysis of HIF expression levels demonstrated that the expression levels of HIF were significantly up-regulated in the treatment group compared with the untreated group(Figure 3D). These results suggested thatRh2 may activate the MAPK signaling pathway and regulate the expression of downstream HIF transcription factors.

*Rh2 inhibits proliferation, promotes apoptosis and inhibits tumor angiogenesis in HepG2 cells*

**Figure 4:**
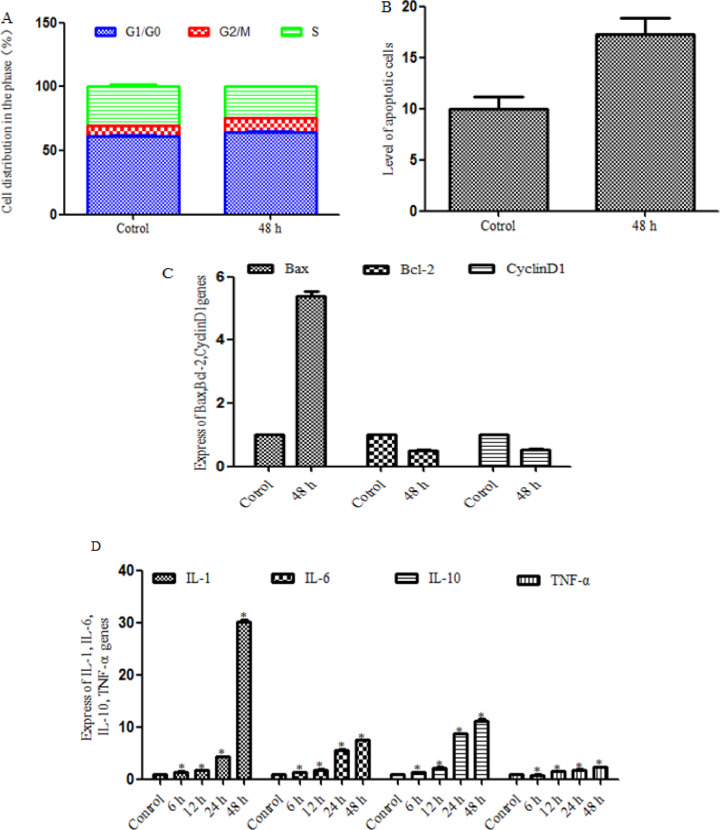
Rh2 Inhibits Proliferation, Promotes Apoptosis and Down-Regulates the Expression Levels of mRNAs Associated with Tumor Angiogenesis in HepG2 Cells. (A) HepG2 cell cycle distribution was analyzed following treatment with Rh2 for 48h. Flow cytometry was used to determine the cell cycle distribution. Blue areas represent the G0/G1 phase, red areas represent the G2/M phase and green areas represent the S phase. Percentage of cell cycle distribution is presented as the mean ± SD. (B) Annexin V-FITC/PI double staining was performed to analyze the levels of apoptosis in HepG2 cells induced byRh2 for 48 h. (C and D) Reverse transcription-quantitative PCR was performed to analyze the mRNA expression levels of Bax, Bcl-2, cyclinD1,IL-1, IL-6, IL-10 and TNF-α. All experiments were repeated at least three times.*P<0.05 vs. control. Rh2, 20(S)-ginsenoside Rh2; PI, propidium iodide; IL, interleukin

HepG2 cells were treated with Rh2 for 48h and the effect ofRh2 on the cell cycle, apoptosis and the expression of genes associated with tumor angiogenesis were analyzed. Flow cytometric analysis of the cell cycle revealed that the percentage of cells in the G0/G1 phase in the treatment group(64.35±1.36%) was significantly increased compared with the untreated group(61.61±1.23%), which suggested that proliferation was inhibited in the treatment group (Figure 4A). Furthermore, to determine the underlying pharmacological mechanisms of Rh2, the expression levels of cell cycle-related genes were determined. The results revealed that the expression levels of CyclinD1 were down-regulated in the treatment group, which may be associated with the G0/G1 cell cycle arrest (Figure 4C).Subsequently, Annexin V/PI double staining was performed to determine the levels of apoptosis. The results revealed that the apoptotic rate was significantly increased in the treatment group compared with the untreated group (Figure 4B). To further validate the effect on apoptosis, RT-qPCR analysis was used to analyze the expression levels of Bax and Bcl-2 following 48h of treatment with Rh2. Compared with the untreated group, the expression levels of Bax were significantly up-regulated, while the expression levels ofBcl-2 were significantly down-regulated in the treatment group, which indicated that Rh2 may induce apoptosis (Figure 4C).Changes in the expression levels of IL-1, IL-6, IL-10 and TNF-α were also determined. As shown in Fig. 4D, the expression levels of IL-1, IL-6, IL-10 and TNF-α were up-regulated in the treatment group, which suggested that the up-regulated expression of IL and TNF-α may inhibit invasion and angiogenesis(Figure 4D).Taken together, these results indicated that Rh2 may inhibit proliferation, promote apoptosis and down-regulate the expression of genes associated with tumor angiogenesis in HepG2 cells.

*Rh2 decreases tumor volume and diameter, and inhibits tumor angiogenesis*

**Figure 5:**
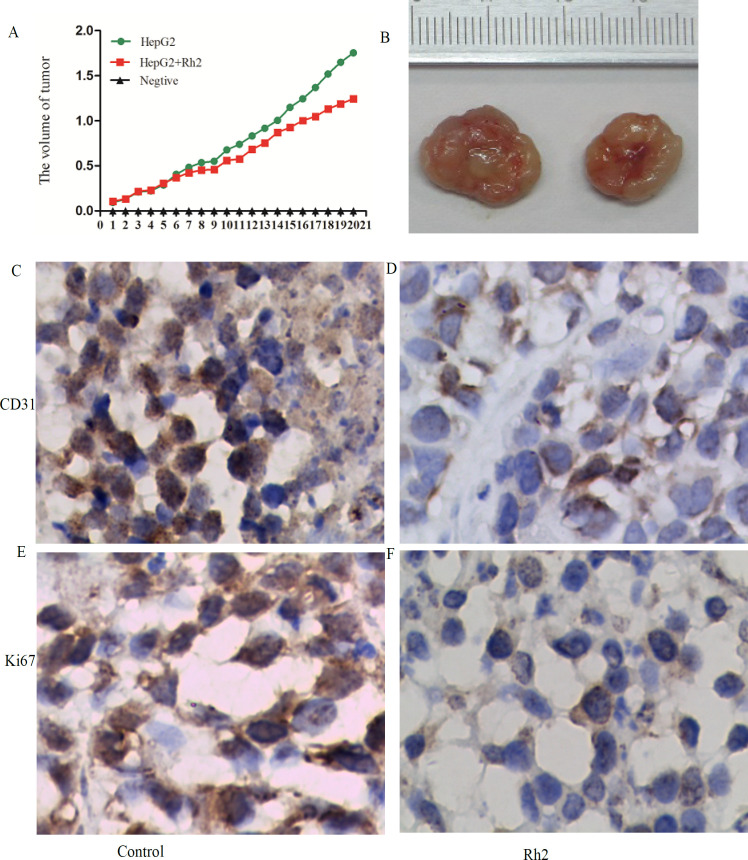
Rh2 Decreases Tumor Volume and Diameter, and Inhibits Tumor Angiogenesis. (A) Mean volume of the tumors from each group is shown. Data are presented as the mean ± SD. (B) Diameter of each tumor from the different groups is shown. (C-F)Immunohistochemical staining was used to analyze the expression levels of CD31 and Ki-67 in the tumor tissue (magnification, x400; scale bar, 200-μm). All experiments were repeated at least three times.Rh2, 20(S)-ginsenoside Rh2

**Figure 6:**
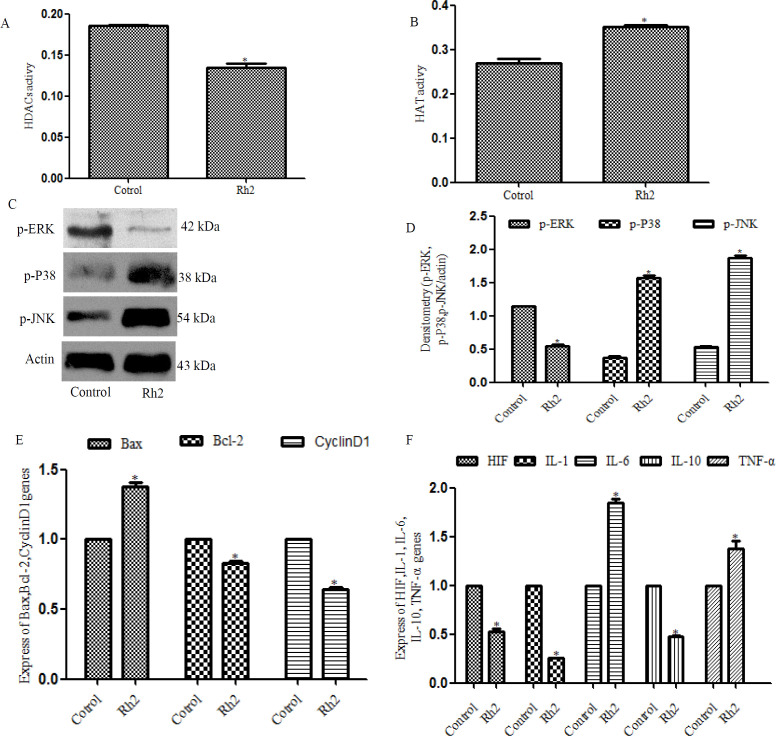
Rh2 Inhibits Total Activity of HDAC and HAT, Activates the MAPK Signaling Pathway and Down-Regulates the Expression Levels of mRNAs Associated with Tumor Angiogenesis in Vivo (A and B) ELISAs were used to analyze the activity of histone deacetylases and histone acetyltransferases in the tumor tissue. (C and D)Western blotting was used to analyze the protein expression levels of p-ERK, p-P38 and p-JNK. (E and F) Reverse transcription-quantitative PCR was used to analyze the mRNA expression levels of Bax, Bcl-2, cyclin D1, IL-1, IL-6, IL-10 and TNF-α. All experiments were repeated at least three times.*P<0.05 vs. control. p-, phosphorylated; IL, interleukin

To determine whether Rh2 exerted similar pharmacological effects in vivo, mice with HepG2 cell-derived xenograft tumors were used in this experiment. The average tumor volume of the treatment group was significantly decreased (1.01±0.134 cm^3^) compared with the control group(1.40±0.113 cm^3^). In addition, the average tumor diameter in the treatment group was significantly lower compared with the control group (Figure 5A and B). Immunohistochemical staining was subsequently used to determine the expression levels of CD31 and Ki-67, which are associated with tumor angiogenesis. The expression levels of both proteins were significantly down-regulated in the treatment group compared with group (Figure 5C-F). In vivo, the HDAC activity in the treatment group was also significantly decreased (Figure 6A). As shown in Figure 6B, the results of the ELISAs demonstrated that total HAT activity was significantly increased in the treatment group. Western blotting analysis revealed that the protein expression levels of p-P38 and p-JNK were significantly up-regulated, while the protein expression levels of p-ERK were down-regulated in the treatment group compared with the untreated group (Figure 6C and D). The expression levels of Bax, IL-1, IL-6, IL-10 and TNF-α were up-regulated, while the expression levels of cyclinD1, Bcl-2 and HIF were down-regulated in the treatment group compared with the untreated group (Figure 6E and F). These results indicated that Rh2 may exert pharmacological effects as a HDAC inhibitor, resulting in decreased tumor volumes and diameters, and the inhibition of tumor angiogenesis.

## Discussion

Previous studies have reported that, ginsenosides, which are used in Traditional Chinese medicine, demonstrated antitumor properties in numerous types of cancer, including malignant liver, gastric and prostate cancer (Tang et al., 2018; Lee et al., 2019; Liu and Fan, 2019; Meng et al., 2019). The results of the present study demonstrated that Rh2, as a HDAC inhibitor, inhibited the proliferation and promoted the apoptosis of HepG2 cells. Rh2 also reduced tumor volume and diameter, and inhibited tumor angiogenesis in a xenograft model. These effects were suggested to occur through the inhibition of HDAC activity, which activated MAPK signaling and down-regulated the expression levels of HIF.

The acetylation levels of nucleosome core proteins and the expression levels of associated genes in human cells were found to depend on the balance between HAT and HDAC activity (Lu et al., 2018a; Freese et al., 2019). The results of the current study revealed that total HAT activity was significantly increased in the treatment group. HATs are known to antagonize the action of HDACs and be associated with the relaxation of the chromatin (Freese et al., 2019), thereby regulating the expression of downstream genes, such as Bax,Bcl-2and cyclinD1.

The observed inhibitory effects of Rh2 over HDACs in HepG2 cells in the present study is consistent with the findings of previous studies. For example, Liu et al., (2015) discovered that Rh2inhibitedthe proliferation, decreased HDAC activity and promoted histone acetylation in K562 cells, which further suggested its role as a HDAC inhibitor.Rg3 also exerted antiproliferative activity in melanoma by reducing the activity of HDACs and increasing p53 acetylation in both experimental and clinical research (Sun et al., 2019). The inhibition of HDAC by Rg3 or short hairpin RNA targeting HDAC could promotec-Jun acetylation, which suppressed proliferation of cutaneous squamous cell carcinoma (CSCC) cells. HDACs were also suggested to represent apotential therapeutic target for CSCC (Zhang et al., 2019a) .

Zhang et al., (2019b) reported that the knockdown ofHDAC6 partially inhibited the growth and migration of colon cancer cells by regulating the MAPK/ERK signaling pathway. Choi et al., (2012) demonstrated that HDAC4 relied on its functional deacetylase domain to activate the MAPK/serpin family F member 2 axis and promote neurogenic muscle atrophy. However, the results of the present study found that the protein expression levels of HDAC1were down-regulated in the treatment group compared with the untreated group, while no significant differences were observed in the protein expression levels of HDAC2 and HDAC6 between the treatment and untreated groups. There are two reasons that may explain the discrepancy between the present results and the aforementioned studies. First, the present study used a different cell line; therefore, discrepancies may exist in the expression levels of HDACs between various cell lines. Second, there are four classes of HDACs, while the present study only analyzed HDAC1, HDAC2 and HDAC6 expression levels.

The findings of the current study revealed that Rh2 could activate the MAPK signaling pathway, which is consistent with the results of previous studies. For example, numerous studies demonstrated that the inactivation of MAPK signaling could inhibit ginsenoside-induced tumor growth and cell migration in colorectal cancer and gastric cancer (Kim et al., 2014; Xie et al., 2018; Liu and Fan, 2019; Lyu et al., 2019). 

HIF plays a vital role in numerous tumor biological processes, including angiogenesis, cellular metabolism, invasion and migration (Masoud and Li, 2015). In the current research, Rh2 was found to down-regulate the mRNA expression levels of HIF by regulating MAPK signaling. This finding is consistent with the findings of Guanet al., (2018), which reported that steroid sulfatase may down-regulate the expression and degrade HIF by suppressing MAPK signaling in cigarette smoke extract macrophages.

Inflammatory ILs and TNF-α have been hypothesized to serve as prospective tumor markers and have been associated with increased tumor invasion and angiogenesis through their ability to inhibit HIF expression (Lee et al., 2018). RT-qPCR analysis in the present study revealed that Rh2 regulated the expression levels of IL-1, IL-6, IL-10 and TNF-α, which inhibited tumor invasion and angiogenesis.

Cell apoptosis is an important cellular pathway that culminates in cell death. Cell apoptosis occurs following the up-regulation of the expression levels of Bax, while cell apoptosis is decreased following the down-regulation of Bcl-2 expression levels. In addition, cell apoptosis also activates caspase-3 and -9, and poly(ADP-ribose) polymerase cleavage (Lu et al., 2018b; Shan et al., 2019). The current results demonstrated that Rh2 promoted the apoptosis of HepG2 cells. Tumor growth has been found to be largely dependent on malignant cell proliferation; therefore, inhibiting cell proliferation has been suggested as an effective method to cure cancer(Cheng et al., 2014; Lyu et al., 2019). Based on these results, it was hypothesized that Rh2 may restrain cellular activity by inducing cell cycle arrest, which may suppress HepG2 cell proliferation and thereby regulate tumor growth.

CD31 and Ki-67are often used as markers of endothelial cell tissue and to assess the levels of tumor angiogenesis (Zhang et al., 2017). The findings of the present study revealed thatRh2 could inhibit angiogenesis in xenograft tumors, which is consistent with the findings of Zhanget al., (2017), which reported that ginsenoside inhibited the growth of breast cancer and glioblastoma by reducing tumor angiogenesis.

Nevertheless, there are several limitations to the present study. First, the study only investigated the effects of the ginsenoside monomer, Rh2; therefore future studies should investigate the effects of other components of ginsenosides to validate the present findings. Second, the present study did not perform gene knocked out to validate the mechanism of Rh2. However, the present experimental results provided preliminarily evidence to suggest the potential pharmacological effects of the ginsenosides monomer, Rh2.

In conclusion, the findings of the present study suggested that Rh2 may exert anticancer effects in HepG2 cells and xenograft tumors by inhibiting HDAC activity and activating the MAPK signaling pathway. These results provided a novel insight into the potential mechanism of Rh2 in liver cancer cells and provided a foundation for the clinical application of Rh2, which is similar to the current application of Rh2 as a Traditional Chinese medicine. 

## Author Contribution Statement

Shi qingqiang made substantial contributions to conception and design, involved in drafting the manuscript. Gao chaoqing, Feng ziqiang and Li jing acquise of data. Qin hongmei and Chen dilong analyze and interpret the data.

## Data Availability

The datasets used during the current study are available from the corresponding author on reasonable request. All data generated or analyzed during this study are included in this published article.
